# Colorimetric Revealing of Ethanol–Water Cluster (E-Wc) Transitions in Binary Solution Based on Starch–I_2_ Crystallization

**DOI:** 10.3390/molecules30183785

**Published:** 2025-09-17

**Authors:** Hui-Shuang Li, Hao-Jie Bai, Hong-Wei Li, Yu-Qing Wu

**Affiliations:** 1 State Key Laboratory for Supramolecular Structure and Materials, College of Chemistry, Jilin University, No. 2699 Qianjin Street, Changchun 130012, China; lihs23@mails.jlu.edu.cn (H.-S.L.); baihj22@mails.jlu.edu.cn (H.-J.B.); lihongwei@jlu.edu.cn (H.-W.L.); 2 Institute of Theoretical Chemistry, College of Chemistry, Jilin University, No. 2 Liutiao Road, Changchun 130023, China

**Keywords:** colorimetry probe of starch–I_2_, ethanol–water cluster (E-Wc), E-Wc transition point, iodine chromogenic effect, starch–I_2_ crystallization

## Abstract

We have developed a highly sensitive colorimetric probe based on starch–iodine (I_2_) crystallization for the precise discrimination of ethanol–water clusters (E-Wc) within binary ethanol–water solutions (E-Ws). This probe enables the identification of specific E-Wc species and their corresponding transition points. Notably, two distinct transition points were identified at ethanol volume fractions of 40–45% and 75–77%. The former corresponds to the structural transition from (H_2_O)_m_(EtOH) to (H_2_O)_m_(EtOH)_n_, characterized by a significant loss of blue coloration, while the latter signifies the transition from (H_2_O)_m_(EtOH)_n_ to (H_2_O)(EtOH)_n_, as evidenced by alterations in the absorption intensity of the starch–I_2_ complex. Mechanistic studies demonstrate that the observed starch–I_2_ crystallization is governed by supramolecular E-Wc rather than individual ethanol or water molecules in the binary solution. By leveraging starch–I_2_ crystallization as a colorimetric bridge, we establish a direct correlation between E-Wc transitions and the iodine chromogenic effect. This approach enables the visual detection of transitions in colorless supramolecular assemblies, offering new insights into supramolecular science. Furthermore, as a simple, rapid, and visually interpretable detection method, this colorimetric probe holds promising applications in fields such as the food industry and supramolecular science.

## 1. Introduction

Supramolecular assemblies and clusters formed through non-covalent interactions (e.g., hydrogen bonds, π-π stacking, hydrophobic effects) demonstrate remarkable potential in materials science, biosensing, and drug delivery. However, colorless supramolecular assemblies/clusters are particularly challenging to study because their transparent nature makes it difficult to directly observe state transitions in solution. This absence of visible optical signals complicates the investigation of their dynamic behavior and functional mechanisms.

Research indicates that the hydrogen bonding (HB) configurations in ethanol–water mixtures exhibit significant differences compared to those observed in pure water or pure ethanol, owing to the formation of distinct E-Wc species that occurs in such binary systems [1]. Concurrently with the dynamic processes of HB formation and dissociation, the structural complexity of E-Wc undergoes substantial variations [2]. Consequently, elucidating the patterns of E-Wc in such binary mixtures is essential for advancing the understanding of ethanol–water systems across diverse application domains. To achieve this objective, a comprehensive suite of experimental techniques has been employed, including mass spectrometry [3,4], Raman spectroscopy [5,6], nuclear magnetic resonance (NMR) spectroscopy [7], neutron diffraction [8,9,10], X-ray diffraction (XRD) [4,11], and infrared spectroscopy [7,12,13], to investigate the molecular interactions between ethanol and water. Furthermore, quantum chemical calculations have also been increasingly adopted as complementary methods to these experimental approaches [14,15,16,17], providing valuable insights into the alterations of HB interactions between ethanol and water molecules.

Compared to conventional experimental approaches that often entail inherent complexity, time-consuming procedures, and significant costs, optical probes offer notable advantages characterized by operational simplicity, rapid response times, high analytical precision, and exceptional sensitivity to molecular environmental changes. These distinctive features make optical probes particularly suitable for tracking hydrogen bonding configuration changes through their responsive behavior to molecular environmental variations. However, the currently reported fluorescent probes can only detect the water content in ethanol or bioethanol by affecting their absorption and emission characteristics through sensitivity to solvent polarity or hydrogen bonding interactions with water molecules [18,19]. To date, no research has documented the application of fluorescent probes for elucidating hydrogen bonding pattern variations in E-Wc systems. Furthermore, the potential utilization of straightforward colorimetric probes in this specific research domain remains entirely unexplored. Given these research gaps, the development of a direct, user-friendly, and visually interpretable colorimetric probe—despite the numerous technical challenges that persist—holds considerable scientific significance and practical value for both fundamental research and potential applications.

Starch functions as the principal carbohydrate constituent in cereal grains and represents a vital nutritional resource for humans. Chemically, it consists of two distinct glucose polymers: amylose, the minor constituent, which exhibits a predominantly linear molecular architecture with molecular weights spanning from 10^5^ to 10^6^, and amylopectin, the predominant component, characterized by its highly branched chain structure with molecular weights ranging between 10^7^ and 10^8^ [20]. The linear amylose molecules typically adopt a helical conformation stabilized by intramolecular hydrogen bonds (HBs) [21]. Upon interaction with iodine molecules (I_2_), starch forms a distinctive helical complex through non-covalent interactions. This starch–I_2_ complex is formed via the accommodation of I_2_ within the hydrophobic cavities of the starch helix, resulting in a thermodynamically stable configuration with notable optical properties. A particularly remarkable characteristic of this complex is its intense colorimetric response, typically manifesting as a deep blue hue. This phenomenon arises from the non-covalent interaction between starch and I_2_, which facilitates charge transfer to generate triiodide ions (I_3_^−^), thereby producing the characteristic blue coloration [22]. The distinct colorimetric behavior of the starch–I_2_ complex has established it as a widely utilized optical probe for analyzing the structural features of starch in aqueous solutions. Previous studies have demonstrated that in middle-concentration ethanol–water systems (48–68 vol%), amylose can form single, left-handed helical inclusion complexes with ethanol, referred to as V-type crystals [23]. More recent investigations have revealed that in 70 vol% E-W systems, starch gelatinization can be entirely suppressed following annealing treatment, with V-type complex formation between starch and ethanol initiating at temperatures exceeding 110 °C [24]. Thus, the incorporation of ethanol into aqueous solutions not only inhibits starch granule swelling but also promotes the formation of V-type single helical complexes, thereby stabilizing starch chains [25]. Consequently, annealing starch in E-W systems has been employed to improve its thermal, shear, and acid resistance, broadening its applicability in the food industry [26].

In general, molecular-level interactions between starch and ethanol primarily involve HBs, hydrophobic interactions, and van der Waals forces [26]. However, in fact, the underlying mechanisms are considerably more complex, as ethanol molecules in E-W systems are never isolated but instead form various E-Wc through complexation with water. The intrinsic mechanism behind it has not been truly solved; in particular, the mechanism between starch and E-Wc and their effects on starch crystallinity have never been investigated. This study aims to address this knowledge gap by employing the starch–I_2_ complex as an optical probe to elucidate the transition behavior of E-Wc. The starch–I_2_ complex, renowned for its sensitivity and distinct colorimetric response, serves as an effective tool for monitoring subtle structural changes within E-Ws. Unlike conventional methods that often rely on indirect measurements or complex theoretical models, our approach leverages the unique optical properties of the starch–I_2_ complex to directly track alterations in the local environment of E-Ws. The transitions of E-Wc in different E-Ws are visually demonstrated through the color changes of the starch–I_2_ complex following thermal-annealing treatments in various E-Ws media. The crystalline structures of starch formed within E-Ws were further characterized employing advanced analytical techniques, including XRD, scanning electron microscopy (SEM), and Fourier-transform infrared spectroscopy (FT-IR), thereby enabling a comprehensive elucidation of the underlying responsive mechanisms. This integrated approach effectively bridges the gap between macroscopic observations and atomic-scale insights. Notably, the starch–I_2_ complex facilitates the correlation of optical property changes with specific structural rearrangements in E-Ws, thereby providing a more refined understanding of their behavior. This methodology establishes a novel pathway for demonstrating transitions in colorless supramolecular assemblies through the use of readily accessible optical probes. Furthermore, it offers a unique capability to visualize and quantify dynamic changes in E-Ws in real time, presenting a new paradigm for investigating their behavior.

## 2. Results

### 2.1. The Colorimetry Responses of the I_2_/I^−^ System to Starch Crystals Formed in Various Ethanol–Water Solutions (E-Ws)

**Figure 1:**
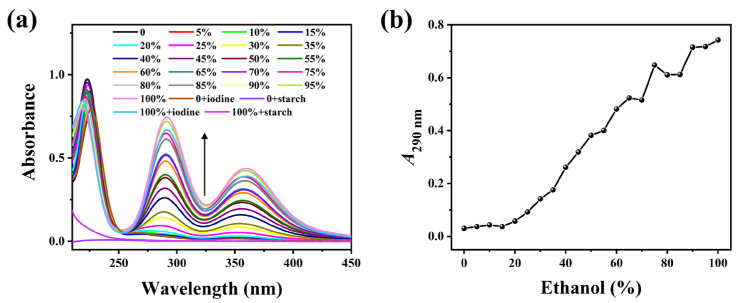
(**a**) Absorption spectra of the mixture of soluble starch and I_2_/I^−^ in different ethanol–water solutions (0–100 vol%) without heat treatment. (The arrow indicates the trend of spectral intensity change.) (**b**) Relationship curves of *A*_290_ with ethanol concentrations.

Figure 1 clearly illustrates that the starch solution, without undergoing thermal annealing treatment, was incubated with E-Ws at different concentrations. Following the addition of the I_2_/I^−^ solution, the mixture was further incubated and subjected to centrifugation to remove large particles. The resulting supernatant remained colorless visually. The UV-vis absorption peaks observed at 290 and 360 nm are attributed to the anionic iodine species (I_3_^−^) [27].

**Figure 2:**
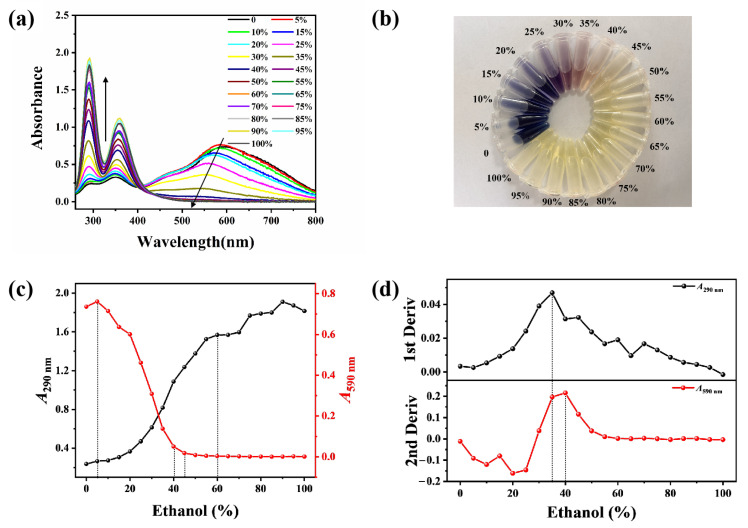
(**a**) UV-vis absorption spectra of the mixture of starch and I_2_/I^−^ in different ethanol–water solutions (0–100 vol%), being prepared based on Approach I. (The arrows indicate the trend of spectral intensity change.) (**b**) Corresponding photos of (**a**) being measured under daylight. (**c**) Relationship curves between *A*_290_ and *A*_590_ with the ethanol concentrations. (**d**) Corresponding first derivative curve of *A*_290_ with ethanol concentrations (upper) and second derivative curve of *A*_590_ with ethanol concentrations (bottom). (The dotted lines indicate the inflection point of the trend change).

Following hydrothermal annealing treatment with varying volumes of E-Ws (Approach I), the introduction of I_2_/I^−^ generates a blue solution exhibiting a broad absorption peak centered at 590 nm (Figure 2a), which is attributed to the formation of the starch–I_2_ complex [26]. As the ethanol concentration increases, the solution color progressively diminishes, transitioning sequentially from blue to purplish-red and ultimately to colorless. Specifically, within the ethanol concentration range of 40–45 vol%, the color evolves from light pink to complete decolorization. Further elevation of ethanol concentration produces negligible additional changes in solution color (Figure 2b). Concurrently, absorption peaks at 290 and 360 nm are observed in the starch-I_3_^−^ aqueous mixture, indicative of free I_3_^−^ species. Notably, the intensities of these peaks exhibit a positive correlation with increasing ethanol concentration, suggesting a reduced formation of the starch–I_2_ complex.

The absorbance values at 290 nm (*A*_290_) and 590 nm (*A*_590_) as functions of ethanol concentration are presented in Figure 2c. With increasing ethanol concentration, *A*_290_ exhibits a continuous upward trend, whereas *A*_590_ demonstrates a corresponding decline. Notably, within the ethanol concentration range of 5–40 vol%, both *A*_290_ and *A*_590_ undergo significant changes: *A*_290_ increases sharply while *A*_590_ decreases markedly. When the ethanol concentration surpasses 45 vol%, *A*_290_ continues to rise rapidly, while *A*_590_ remains relatively stable with minimal variation. At ethanol concentrations exceeding 60 vol%, *A*_290_ displays irregular fluctuation patterns, whereas *A*_590_ maintains remarkable stability (Figure 2c).

The second derivative curve of *A*_590_ values as a function of ethanol concentration exhibits a distinct transition region between 35 and 40 vol%, which corresponds to the inflection point observed at 35 vol% in the first derivative curve of *A*_290_. Notably, when the ethanol concentration surpasses 60 vol%, the curve becomes linear and loses sensitivity to the structural change occurring at 75 vol% (Figure 2d). Consequently, thermal annealing treatments of starch were performed using E-Ws with concentrations ranging from 60–79 vol% (in 2 vol% increments), as outlined in Approach II.

**Figure 3:**
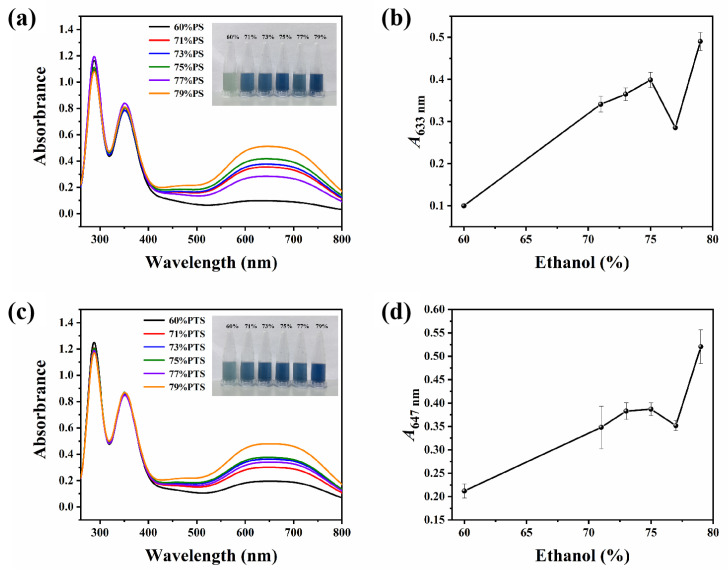
(**a**) UV-vis absorption spectra and the corresponding photos (insert) of the mixture of PS and I_2_/I^−^ under daylight after thermal annealing treatments in 60–79 vol% E-Ws (approach II). (**b**) The plot of the relationship between corresponding *A*_633_ and ethanol concentrations. (**c**) UV-vis absorption spectra and the corresponding photos (insert) of the mixture of PTS and I_2_/I^−^ under daylight after thermal annealing treatments in 60–79 vol% E-Ws (Approach II). (**d**) The plot of the relationship between corresponding *A*_647_ and ethanol concentrations.

As shown in Figure 3, distinct absorption peaks were observed within the wavelength range of 500–800 nm in the PS and I_2_/I^−^ mixture, indicating the formation of an amylose–I_2_ complex (Figure 3a,c). Typically, absorbance values serve as an indicator of the complexation affinity between starch and I_2_. In this interaction, starch chains adopt a single helical conformation, enabling electron transfer between iodine molecules and starch upon entry into the helical cavity, which consequently generates coloration (with the amylose–I_2_ complex exhibiting a blue hue and the amylopectin-I_2_ complex displaying a purple-red coloration) [28]. Thus, a stronger starch–I_2_ interaction corresponds to higher absorbance values in the 500–800 nm range and a more intense blue coloration.

As demonstrated in the inset of Figure 3a, with progressive increases in ethanol concentration, the color of the PS-I_2_ complex transitions from light to dark blue. However, when the ethanol concentration increases from 75 vol% to 77 vol%, an anomalous fluctuation is observed, where both the color intensity and the *A*_633_ value deviate from the previously established increasing trend (Figure 3b). A similar phenomenon was also observed for PTS (Figure 3c,d), further confirming this irregularity in the PS system.

### 2.2. Structure and Morphology Characterizations of Starch Crystal

#### 2.2.1. Long-Range Helical Structures Revealed by X-Ray Diffraction (XRD)

**Figure 4:**
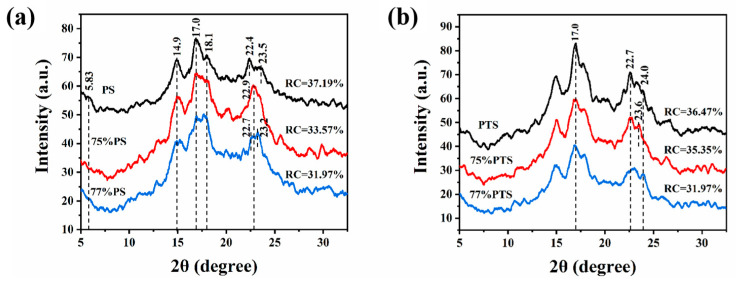
X-ray diffraction curves of (**a**) native pea starch (PS), and (**b**) native potato starch (PTS), and after thermal annealing in 75 vol% (red) and 77 vol% (blue) E-Ws (Approach II), respectively.

XRD is widely utilized to characterize the helical structures of long-range ordered crystals formed in starch. The XRD patterns of PS and PTS before and after thermal annealing in 75 vol% and 77 vol% E-Ws are compared in Figure 4. Initially, prior to thermal treatment (black curves in Figure 4a), PS exhibited a typical C-type crystalline structure [26], characterized by a distinct peak at 5.83°, two peaks at 14.9° and 23.0°, and an additional pair of peaks at 17.0° and 18.1°. Following thermal annealing, however, the peak at 5.83° nearly disappeared while the other four peaks remained well-defined, indicating a thermal-induced transition from C-type to A-type crystalline structure [29]. The RC was subsequently calculated based on the integrated area of characteristic diffraction peaks. The results revealed an overall decrease in RC from 37.19% to 33.57% and 31.97% for the two starch samples after thermal annealing in E-Ws.

Moreover, the XRD analysis unveils several significant structural modifications (Figure 4a). The peak intensity corresponding to starch at 14.9° exhibited a marked reduction following thermal annealing treatment, concomitant with a broadening of the peak width. Simultaneously, the peaks at 17.0° and 18.1° progressively widened, accompanied by a decrease in their relative intensity ratio. Furthermore, the peak morphology near 23.0° became sharper, while its intensity displayed fluctuations post-annealing. These observations provide compelling evidence of the structural alterations in starch crystallinity induced by thermal annealing.

Additionally, based on Equation (1), the relative RC of PS in 77% E-Ws (31.97%) was lower than that in 75% E-W solution (33.57%). This difference can be attributed to variations in starch granule gelatinization during thermal treatment [26], leading to greater fracture of the crystalline domains formed during the annealing process in the 77% E-W system.

As illustrated in Figure 4b, in contrast to the untreated sample, the annealed PTS specimen exhibited a typical B-type crystal structure, which is commonly observed in native PTS, indicating negligible changes in crystallinity. Notably, thermal annealing treatment resulted in a decrease in peak intensity and an increase in peak width at 15.0°, 17.0°, and 22.7°, along with fluctuations in peak intensity near 24.0°. These changes enabled the evaluation of RC for PTS before and after thermal annealing in systems containing either 75 vol% or 77 vol% E-Ws. Based on Equation (1), the RC value of PTS in the 77 vol% E-Ws system (31.97%) was significantly lower than that in the 75 vol% E-Ws system (35.35%), which is in excellent agreement with the previously observed trends for PS.

#### 2.2.2. Granular Morphologies of Starches Revealed by SEM Images

**Figure 5:**
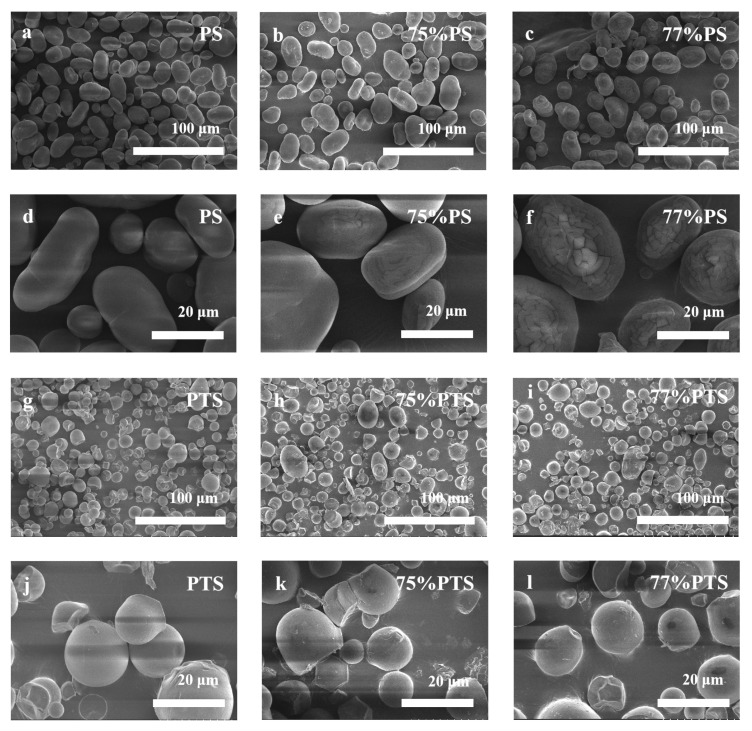
SEM images of (**a**,**d**) native PS and thermal annealing-treated PS by (**b**,**e**) 75 vol%, (**c**,**f**) 77 vol% E-Ws; (**g**,**j**) native PTS and thermal annealing-treated PTS by (**h**,**k**) 75 vol%, (**i**,**l**) 77 vol% E-Ws (Approach II) at different scales, respectively.

The morphological features and surface characteristics of native PS and thermally annealed PS are presented in Figure 5. Native starch granules exhibit a smooth surface devoid of cavities or fractures, with shapes varying from kidney-shaped and spherical to oval and displaying a broad size distribution. Smaller granules predominantly adopt a spherical morphology, whereas larger granules tend toward an oval configuration [30]. The kidney shape, however, remains a consistent morphological feature across all size ranges (Figure 5a,d).

Thermal annealing treatment induces only minor surface disruptions without significantly altering the overall granule morphology. While some granules develop indentations or minor fractures following annealing, the primary structural integrity is preserved. Comparative analysis between PS samples in 75 vol% E-Ws (Figure 5b,e) and those in 77 vol% E-Ws (Figure 5c,f) reveals that the latter exhibit deeper and more pronounced surface cracks. This difference can be attributed to the greater degree of partial swelling and gelatinization experienced by starch granules during annealing in the 77 vol% E-Ws, followed by shrinkage during the drying process [31]. Notably, the 77% PS system undergoes more extensive swelling compared to the 75% PS system, leading to the formation of deeper and larger fractures.

Native PTS granules showed smooth surfaces with truncated, circular shapes (Figure 5g,j). After E-Ws thermal annealing, PTS granules developed surface indentations and small fractures, exhibiting roughened surfaces and occasional irregular, blocky structures. The comparison between 75% PTS (Figure 5h,k) and 77% PTS (Figure 5i,l) demonstrated deeper cracks and more prevalent blocky structures in the 77% PTS samples. Notably, the morphological changes in PTS following thermal annealing closely mirrored those observed in PS under identical treatment conditions.

#### 2.2.3. Fine Structural Differences in Native and Annealed Starch Samples Revealed by FT-IR Spectroscopy

**Figure 6:**
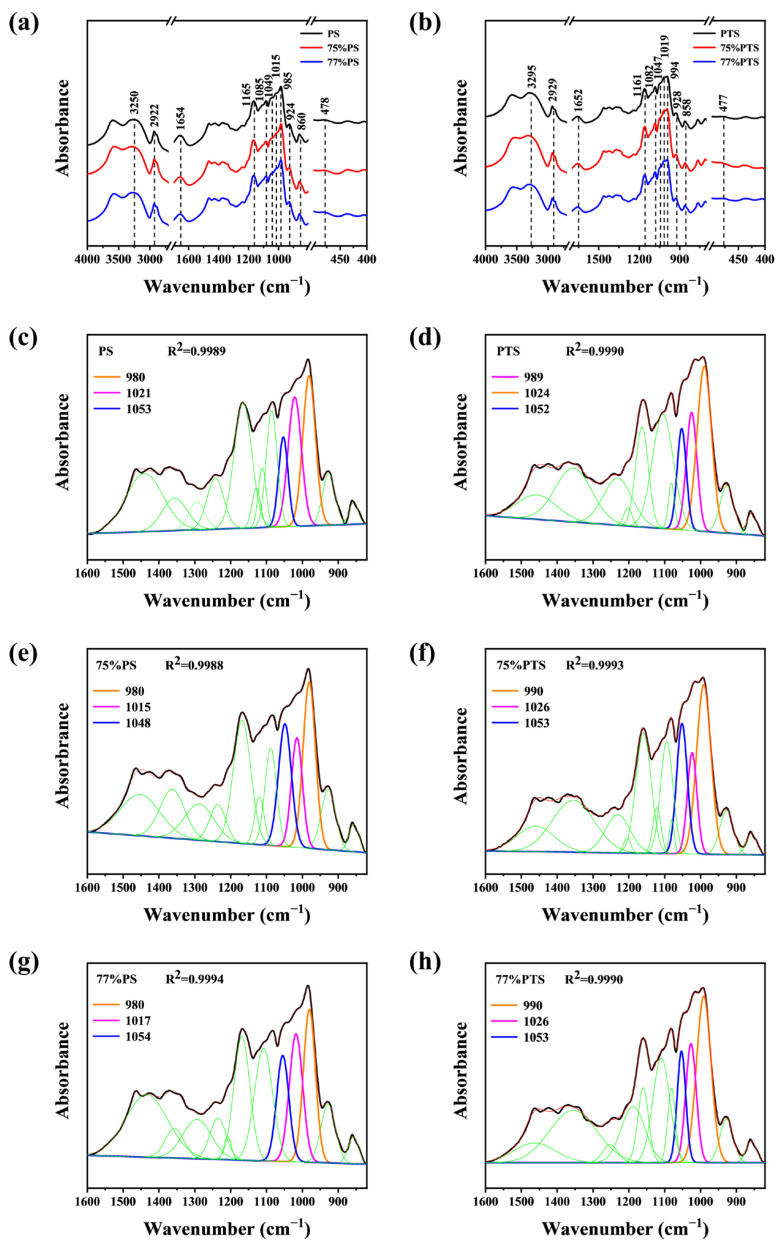
FT-IR patterns of native and thermal annealing-treated (**a**) PS, and (**b**) PTS by E-Ws. Split-peak fitting curve of (**c**) native PS, (**d**) 75 vol%, and (**e**) 77 vol% E-Ws-treated PS, and (**f**) native PTS, and (**g**) 75 vol% (**h**) 77 vol% E-Ws-treated PTS (Approach II). (The black lines in panels (c) to (f) represent the experimental infrared data of the sample, while the green lines correspond to 9 distinct peaks obtained from Gaussian deconvolution within the spectral region of 1600–820 cm^−1^, excluding the peaks at 1047, 1022, and 995 cm^−1^).

FT-IR spectroscopy is a crucial method for analyzing the composition and structure of samples. It allows for the determination of ligand complexation with starch and helps identify structural differences between samples. Using FT-IR spectroscopy, detailed investigations were carried out on the material composition, conformation, configuration, and crystalline structure of starch samples. The results obtained from the analysis of native and annealed starch samples are presented in Figure 6 for comparison.

All samples, including both native starch and those subjected to thermal annealing in the E-Ws, exhibited a broad absorption band at ~3250 cm^−1^, attributed to abundant O-H groups in starch molecules. A minor absorption peak at 2922 cm^−1^ was assigned to C-H stretching vibrations [32]. Additionally, the peak near 1654 cm^−1^ corresponds to the vibrational frequency of the aldehyde group (C=O) in starch. Absorption bands at 1165 cm^−1^ and 1085 cm^−1^ were observed in all samples, corresponding to C-O stretching vibrations. Furthermore, the absorption peak at ~985 cm^−1^ indicates C-OH vibration, associated with intramolecular hydrogen bonds at the C6 position of starch [33,34]. Besides, characteristic bands at 478 cm^−1^, 860 cm^−1^, 924 cm^−1^, and 2922 cm^−1^ were linked to CH_2_, C_1_-O-C_4_, C_1_-O-C_5_, and C-H vibrational modes, respectively [35].

The remarkable similarity evident in the FTIR spectra across all samples indicates that the short-range ordering of the annealed PS samples closely resembles that of native amylose starch. Likewise, thermal annealing of PTS resulted in a short-range molecular arrangement that aligns closely with that of natural PTS, as corroborated by X-ray diffraction analysis [36]. Nevertheless, subtle structural distinctions between these materials can be effectively identified through subsequent spectral split curve-fitting analyses.

The split-peak fitting results for both native and annealed samples in the 1600–820 cm^−1^ spectral region are presented in Figure 6c–h, respectively. Gaussian deconvolution revealed 12 distinct peaks within this wavenumber range, with peak intensities quantified by their corresponding peak areas. Taking native PS as an example, the absorption bands at 1049 cm^−1^ and 985 cm^−1^ are attributed to the crystalline regions, while the band at 1015 cm^−1^ corresponds to the amorphous regions of starch. Consequently, the short-range ordered helical structures of starch can be effectively characterized by analyzing the area ratios of 1049/1015 and 1015/985, as previously reported [37]. Specifically, higher 1049/1015 ratios and lower 1015/985 ratios indicate a more ordered short-range internal structure [24].

Following peak deconvolution, the calculated area ratios are summarized in Table 1. Comparative analysis with the native PS spectrum demonstrated significant increases in both the 1049/1015 and 1015/985 absorbance ratios for PS starch, suggesting enhanced short-range helical ordering. Furthermore, when compared to the 75%PS sample, the 77%PS exhibited a reduced degree of short-range ordering, which is in good agreement with the XRD findings.

Thermal annealing treatment similarly induced an increase in the short-range helical structure of PTS. Similarly, the short-range helix of 77%PTS was lower than that of 75%PTS, illustrating again the gelatinization difference of starch granules during the thermal process [26], resulting in more fractures of the crystalline granules during annealing of 77%PS.

## 3. Discussion

### 3.1. Small Molecule Interference on Amylose Crystallinity

Starch consists of a mixture of amylose and amylopectin molecules. The linear segments of amylose chains and the side chains of amylopectin can form complexes with various small molecules, including iodine, alcohols, fatty acids, aromatic compounds, salicylic acid, and even ibuprofen [38]. Depending on environmental conditions, amylose in aqueous solution may adopt a left-handed helical conformation or exist as random coils. Notably, when amylose is in a single-helical state, its interior exhibits greater hydrophobicity than its surface [39]. Upon thermal treatment of starch, numerous single helices can interact with hydrophobic guest molecules and subsequently reassemble into new V-type crystalline structures. Thus, the preformation of V-type starch serves as a promising strategy for accommodating small guest molecules via hydrophobic interactions and diffusion driven by concentration gradients [40].

Chen et al. employed the characteristic color reaction between starch and iodine to evaluate the complexation affinity between starch and ethanol. Their findings suggest that when ethanol molecules occupy the helical cavity of amylose, the available binding sites for iodine–amylose interaction are reduced, thereby diminishing the intensity of the color reaction [26]. This phenomenon indicates that ethanol–starch complexation alters the crystalline structure of amylose, consequently reducing its iodine-binding capacity. Moreover, through systematic investigation of the amylose-I_2_/I^−^ system, they observed subtle shifts in the absorption maximum (~600 nm) under varying conditions, including different iodide concentrations and amylose chain lengths modified by amylase treatment [41].

### 3.2. Macromolecule Interference on the Amylose Crystallinity

In addition to small-molecule compounds, macromolecular substances, including proteins, cellulose, and pectin, can also interact with starch to form complexes. These interactions can substantially modify the physicochemical properties and structural characteristics of the starch system, including viscosity, dynamic modulus, gel enthalpy, and even crystallinity [42,43,44]. The formation of starch–guest complexes is predominantly governed by non-covalent interactions, encompassing van der Waals forces, HBs, and hydrophobic interactions. Among these, hydrophobic interactions play a particularly pivotal role, as the helical cavities formed by amylose chains exhibit inherent hydrophobicity. Notably, the internal hydrophobic environment of the helix enables the binding of non-polar regions of guest molecules through such non-covalent interactions. Concurrently, the polar regions of the guest molecules can establish HBs with the hydroxyl groups located on the outer surface of the helix.

Furthermore, due to the large molecular size of guest compounds, steric hindrance can significantly influence the formation of starch helical structures. For instance, to examine the effects of high-pressure homogenization (HPH) on starch structure, the honokiol (HK) and arctiin (AC), were selected as representative model compounds of lignans [45]. Under high-shear conditions, lignans were observed to form complexes with starch molecules via hydrophobic interactions or HBs, potentially inducing amylose to form double helices. These complexes could further assemble into crystalline structures and ultimately become entangled into aggregates.

Moreover, it was observed that starch subjected to HPH treatment exhibited a reduced double helical structure when combined with AC compared to HK. Besides, upon the addition of the same lignan, HK demonstrated a greater propensity to form V-type crystallinity than AC. These results indicate that the smaller molecular size of HK facilitates its penetration into the hydrophobic cavity of amylose, thereby promoting the formation of a single helix. This, in turn, enables the amylose molecules to arrange more regularly into a double helical structure due to the relatively weak steric hindrance. In contrast, AC encountered greater difficulty in forming an inclusion complex with amylose or inducing the self-assembly of starch molecules, as its larger molecular size resulted in stronger steric hindrance during complexation. Consequently, this led to a reduction in starch crystallinity when AC was employed.

### 3.3. Interference of Supramolecular Complex with Starch Crystallinity

It is widely established that pure liquid water forms a dynamic three-dimensional (3D) hydrogen-bonded network [46]. Consequently, the introduction of a small fraction of alcohol into water typically disrupts the intermolecular HBs among water molecules, resulting in an endothermic process. Surprisingly, however, experimental observations reveal that the mixing of water and alcohol generates excessive heat evolution [46]. As early as 1945, Frank and Evans proposed an explanation for the observed negative excess entropies and enthalpies in E-W systems, attributing these phenomena to the formation of an “iceberg” structure around the hydrophobic groups of alcohol molecules in solution [47]. This hypothesis has since motivated extensive research into various molecular association mechanisms, including clathrate-like structures, in E-W mixtures. Nevertheless, a substantial body of both experimental and theoretical investigations has demonstrated that ethanol–water mixtures actually form percolating 3D networks. In this structural model, ethanol and water molecules are interconnected through hydrogen bonding to establish a dynamic and percolating network architecture. Notably, this network exhibits a “percolation threshold” within a specific concentration range: when the molar fraction of either component reaches a critical value, its HB network abruptly becomes continuous and assumes dominant control over the structural organization of the system [8,48,49].

In addition, these mesoscopic clathrate structures play a wide range of roles in aqueous solutions and exert a significant influence on the HB network of water molecules [46]. Given the significance of E-W mixtures across various fields, researchers have conducted detailed investigations into the different supramolecular E-Wc present in this binary system. They used fluorescence spectroscopy and 2D correlation analysis at different volumes of E-W mixtures and incubation times after mixing [50]. Through 2D correlation analysis of E-W mixtures with ethanol concentrations ranging from 10 vol% to 100 vol% at each incubation time, it was found that the correlation intensity of the emission peak at 330 nm for (H_2_O)_m_(EtOH)_n_ not only correlated with ethanol concentration but also increased gradually with incubation time. These findings suggest that the (H_2_O)_m_(EtOH)_n_ cluster presented is the most stable one, as it tends to form over longer incubation periods. Moreover, the correlation results indicate that in the low-concentration range of 10–45 vol%, the dominant cluster is the hydrophilic (H_2_O)_m_(EtOH), corresponding to an emission peak at approximately 373 nm. Conversely, in the high-concentration range of 80–99 vol%, the predominant cluster is the hydrophobic (H_2_O)(EtOH)_n_, corresponding to an emission peak at approximately 310 nm. However, the most stable cluster of (H_2_O)_m_(EtOH)_n_, corresponding to an emission peak at 330 nm, not only predominates in the concentration range of 50–75 vol% but also exists in all E-W mixtures after longer incubation times.

Additionally, researchers employed Raman spectroscopy, Fourier transform infrared-attenuated total reflection (FTIR-ATR), terahertz time-domain spectroscopy, and pulsed-field gradient nuclear magnetic resonance (PFG-NMR), combined with molecular dynamics simulations and density functional theory, to deduce the possible structures of ethanol–water clusters at different concentrations [51,52,53,54,55]. The results indicate that in the water-rich region at low ethanol concentrations, where the concentration of water exceeds that of ethanol, the interactions between water molecules are stronger. Thus, the primary clusters formed are (H_2_O)_m_(EtOH), with ethanol molecules embedded within cage-like clusters (H_2_O)_m_ composed of numerous water molecules. The hydrogen bond strength between ethanol and water reaches its maximum at a 40 vol% ethanol concentration. As the ethanol concentration increases further, the hydration structure becomes less stable, the hydrogen bond strength between ethanol and water weakens, and ethanol molecules begin to self-associate. They form (H_2_O)_m_(EtOH)_n_ clusters with water molecules through hydrogen bonding, which are closed or open planar ring-like clusters with high rigidity, where water molecules bridge ethanol molecules via hydrogen bonds. In the high ethanol concentration region, ethanol molecules preferentially self-associate to form short-chain molecular clusters, while water molecules primarily engage in hydrogen bonding with the hydrophilic hydroxyl groups of these ethanol clusters, forming E-Wc-type structures represented as (H_2_O)(EtOH)_n_.

**Figure 7:**
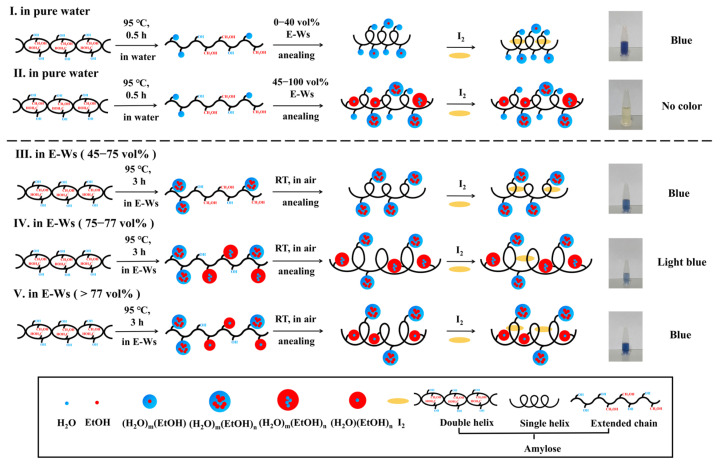
The mechanism of color changes showing for starch–I_2_ along with thermal-heating treatments in different vol% of E-Ws.

In summary, both small and large molecules can form complexes with starch via HBs, van der Waals forces, and/or hydrophobic interactions. Generally, as the concentration of small molecules increases, a greater number of complexes are formed with starch. However, for macromolecules, steric hindrance effects make their complexation with starch more challenging. Molecules with relatively small steric hindrance can arrange into a regular double-helical structure, whereas those with greater steric hindrance have difficulty forming inclusion complexes with starch or effectively inducing starch self-assembly [45]. In this study, after subjecting starch to thermal annealing treatments under varying E-W ratios, the subsequent addition of iodine induced distinctly different color responses in the treated samples. Notably, when the ethanol concentration increased from 75 vol% to 77 vol%, the blue color of the complex became noticeably lighter, and the absorbance value at 633 nm (*A*_633_) did not exhibit a gradual decline with rising ethanol concentrations. Based on these observations, we further investigated the influence of supramolecular complexes on starch crystallinity, as illustrated in Figure 7.

### 3.4. Intrinsic Mechanism of Starch–I_2_ to Show Different Colors

#### 3.4.1. Phase Transition Behavior of 40–45 vol% in E-Ws?

The hydrophilic hydroxyl groups of α [1→4] glucan helices are predominantly located on the exterior surface, while the hydrophobic hydroxymethyl groups and oxygen atoms of the glycosidic bonds reside within the interior, forming a hydrophobic cavity capable of encapsulating hydrophobic ligands. This structural arrangement establishes the amylose helix as a hydrophobic interior with a hydrophilic exterior [56].

Amylose is known to form crystalline inclusion complexes with both polar and non-polar compounds, exhibiting a well-defined V-type amylose helical structure. Early X-ray diffraction studies demonstrated that the helical conformation of this polysaccharide is strongly influenced by the size of the complexing agent. Specifically: (i) linear alcohols and fatty acids induce the formation of 6-fold helices (6 D-glucosyl residues per turn); (ii) Branched-chain alkyl compounds, such as tert-butyl alcohol, promote 7-fold helices (7 D-glucosyl residues per turn); (iii) bulky molecules like 1-naphthol result in 8-fold helices (8 D-glucosyl residues per turn) [57].

Furthermore, iodine molecules readily incorporate into the hydrophobic cavity of the amylose helix via van der Waals interactions, forming a characteristic left-handed single-chain helical complex [58]. This complex typically exhibits an outer diameter of ~13 Å and a pitch of 8 Å (with six 1,4-glucose units per pitch), hosting an internal cavity of ~5 Å, where the iodine alongside iodide is accommodated with I-I distances of ~3.1 Å [41]. The subtle variations in the blue coloration of starch–I_2_ complexes observed in different E-W systems can be attributed to differences in the E-Wc size and its phase transition behavior within the 40–45 vol% E-W range.

The proposed mechanisms governing the color evolution of starch–I_2_ complexes in pure water and various E-W mixtures are schematically illustrated in Figure 7. When starch granules are heated in pure water, they undergo hydration-induced swelling, ultimately leading to the disruption of both granular and crystalline structures at elevated temperatures (95 °C). This thermal treatment facilitates the dissociation of the double helical structure into extended single chains. Subsequent interactions between water molecules (or their clusters) and hydroxyl groups via non-covalent forces, combined with annealing and I_2_/I^−^ incorporation, promote the formation of single helices that bind to I_2_, resulting in the characteristic blue coloration.

In contrast, the introduction of varying ethanol concentrations in the annealing medium alters this process. Ethanol forms E-Wc within the mixture, which significantly influences the final structural configuration. The resulting single-stranded helices may or may not bind to I_2_, depending critically on the size and physicochemical properties of the E-Wc formed under different ethanol–water compositions.

(I) When the ethanol concentration is below 40 vol%, the E-W system is primarily composed of (H_2_O)_m_(EtOH) clusters, where ethanol molecules are embedded within cage-like (H_2_O)_m_ clusters formed by numerous water molecules, exhibiting internal hydrophobicity and external hydrophilicity [51,52,53,54,55]. Upon heating at 95 °C in aqueous solution followed by annealing in such E-W systems, non-covalent interactions occur between these clusters and the hydroxyl groups of liberated amylose. These interactions do not hinder the formation of single-helix amylose structures or their encapsulation of I_2_ within the helical cavity, resulting in a characteristic blue solution, as illustrated in Figure 7 (I).
(II) When the ethanol concentration ranges from 45 to 75 vol%, larger and more structurally complex (H_2_O)_m_(EtOH)_n_ clusters predominantly form in the E-W system. Under these conditions, the clusters may interact with either the hydrophobic or hydrophilic regions of the amylose chain and localize either on the exterior or interior of the helix following annealing. However, due to steric constraints imposed by the E-W system, the iodine–helix interaction is likely attenuated, leading to partial or complete decolorization of the blue coloration, as illustrated in Figure 7 (II).

#### 3.4.2. Phase Transition Behavior of 75–77 vol% in E-Ws?

(III) Within the ethanol volume fraction range of 45–75%, the predominant aggregates in E-Ws exist as (H_2_O)_m_(EtOH)_n_ (m > n) configurations. Owing to the persistent exposure of hydroxyl groups from water molecules, these aggregates maintain pronounced hydrophilic characteristics. During the thermal treatment of starch in E-Ws, as temperature progressively increases, amylose initially dissolves and leaches from the starch granules. At this stage, supramolecular E-Wc engages in interactions with the extended starch chains, facilitating the formation of a composite structure.

Following room-temperature annealing in E-Ws, the cluster of (H_2_O)_m_(EtOH)_n_ remains stably adsorbed onto the exterior surface of the single helix. This spatial arrangement not only permits uninterrupted iodine access but also sustains the characteristic blue coloration of the solution, as illustrated in Figure 7 (III).

(IV) When the ethanol concentration rises to 75–77 vol%, the E-Ws systems contain both hydrophilic and hydrophobic (H_2_O)_m_(EtOH)_n_ clusters. These clusters can selectively interact with the hydrophilic hydroxyl groups and hydrophobic hydroxymethyl groups of the α [1→4] glucan helix through specific recognition mechanisms. During the annealing process, a composite structure forms where hydrophilic clusters localize on the outer surface of the helix, while hydrophobic clusters embed within the helical cavity. This structural reorganization of starch helices may lead to an increase in the helical pitch [59]. Simultaneously, the larger molecular dimensions of (H_2_O)_m_(EtOH)_n_, compared to (H_2_O)(EtOH)_n_ [60], not only augment the overall helix diameter but also attenuate its binding affinity with iodine molecules. Ultimately, the diminished formation of starch–I_2_ complexes leads to a noticeable weakening of the blue coloration in the solution, as illustrated in Figure 7 (IV).
(V) When the ethanol concentration exceeds 77 vol%, the hydrophobic clusters (H_2_O)_m_(EtOH)_n_ (m < n) in solution reorganize into an externally hydrophobic and internally hydrophilic structure, (H_2_O)(EtOH)_n_ [51]. Compared to the original (H_2_O)_m_(EtOH)_n_ clusters, the reduced size of (H_2_O)(EtOH)_n_ leads to a decrease in helix spacing during amylose chain helix formation. Upon iodine binding, the interaction sites between iodine and the hydrophobic spiral cavities are re-established, restoring the characteristic blue complex, as illustrated in Figure 7 (V).

In summary, within E-W systems at varying concentrations, ethanol and water exist predominantly as E-Wc rather than as individual molecules. The distinct structural configurations, sizes, and surface properties of these E-Wc result in differential binding sites and interaction modes with amylose through non-covalent forces, which are governed by the helical pitch and hydrophobic cavity dimensions of the single-helix structure formed after annealing. Further iodine addition induces color changes in the solution, corresponding to the formation of specific starch–I_2_ complexes. Consequently, this phenomenon enables the differentiation of E-Wc across different ethanol–water concentration regimes. We propose that this approach not only facilitates the identification of E-Wc in E-Ws but also holds potential for distinguishing various types of emulsifiers or surfactants under appropriately designed experimental conditions.

## 4. Materials and Methods

### 4.1. Materials

Soluble starch was purchased from Tianjin Guangfu Fine Chemical Research Institute (Tianjin, China). Pea starch (PS), was procured from Xiushan Huolang Food Development Co., Ltd. (Chongqing, China). Potato starch (PTS) was acquired from Hebei Gufu Food Co., Ltd. (Langfang, China). Potassium iodide was obtained from Tianjin Bodi Chemical Co., Ltd. (Tianjin, China). Iodine was sourced from Tianjin Xinbote Chemical Co., Ltd. (Tianjin, China). Absolute ethanol was purchased from Shanghai Aladdin Biochemical Technology Co., Ltd. (Shanghai, China).

### 4.2. Heating in Water and Annealing in E-Ws of Soluble Starch (Approach I)

A total of 100 mg of soluble starch and 1 mL of distilled water were mixed and heated at 95 °C for 30 min. Then, 10 μL of it was mixed with 1 mL of E-Ws (0–100 vol%, with 5 vol% increment) and 40 μL of iodine/iodide solution (I_2_/I^−^; 0.005 mol/L I_2_ and 0.01 mol/L KI) for annealing and complexing with I_2_. After further incubation at room temperature (RT) for 1 h, the mixture was centrifuged, and the supernatant was collected for UV-vis absorption spectral measurement.

### 4.3. Thermal Heating and Annealing of PS and PTS in E-Ws (Approach II)

The thermal heating and annealing treatments of starch in E-Ws were conducted according to an established method [26], with minor modifications. In brief, 12 g of PS or PTS were mixed with 28 mL of E-Ws with different ethanol volumes (vol%), and then placed in sealed containers, which were shaken in a 95 °C water bath for 3 h. After annealing at RT for 1 h, the mixture was centrifuged. Finally, the starch slurry was dried in an oven (45 °C) for 24 h to become solid for the measurements of XRD and FT-IR spectrum. The starch of PS and PTS after being treated with thermal heating and annealing by different volumes of E-Ws is abbreviated as X%PS or X%PTS, where the letter X stands for the volume ratio of ethanol to water.

The sample (0.4 g) and distilled water (4.6 mL) were mixed in a centrifuge tube and heated at 100 °C for 30 min. Twenty-five milliliters of distilled water were added to the centrifuge tube and cooled to room temperature. The mixture (50 μL) was taken out, mixed with distilled water (1.5 mL) and iodine/iodide solution (0.2 mL), and then the spectra of the annealed starch–I_2_ complex were measured with an ultraviolet-visible spectrophotometer at 260–800 nm.

### 4.4. UV-Vis Absorption Spectrum

The maximum absorption wavelength (λ_max_) of native and annealed starches was determined following a previously established method [26] after minor adjustments, both for the native and treated starches achieved in approaches I and II. All the spectra were recorded using a Shimadzu UV-3600 spectrophotometer (Kyoto, Japan) over the optical range of 260–800 nm.

### 4.5. XRD

The samples were conditioned for 48 h in a desiccator containing saturated sodium chloride solution (relative humidity 75%) to stabilize the moisture content at above 20% [61,62]. Subsequently, the crystalline structures of the samples were analyzed using an X-ray diffractometer (D8 Venture, Bruker, Germany) and scanned utilizing Cu-Kα radiation with a scanning speed of 3° per minute between two thetas of 4° and 40°.

The relative crystallinity (RC) of the starch in each sample was determined using Origin software (version 6.5, Materials Data Inc., Livermore, CA, USA), by comparing the integrated area under the specific peaks for crystalline with the area of the total region using the following formula:

RC (%) = CDA × 100/TDA,(1)

where CDA denotes the dispersed area of crystalline, and TDA denotes the total dispersed area.

### 4.6. SEM

The morphology of the starch samples was examined using a scanning electron microscope (SEM, Hitachi Regulus 8100, Hitachi, Japan). To prepare the samples, they were mounted on an aluminum stub using double-sided adhesive tape and then coated with gold palladium using a sputter coater. Subsequently, the stub was positioned under the scanning electron microscope with an acceleration voltage of 10 kV. To gain a detailed understanding of the surface morphology, representative digital images of the samples were captured at magnifications of 500× and 2000×, enabling a closer examination of the intricate features present on the surface.

### 4.7. FT-IR Spectroscopy

The FT-IR spectra of native and treated PS and PTS were obtained using an infrared spectrometer (VERTEX 80V, Bruker, Germany). The solid sample was mixed with spectroscopic-grade KBr and then pressed into tablets. Calibration was carried out by using potassium bromide as a blank. Then, the spectrum was acquired using the software provided by the instrument company. Spectra of the PS and PTS were obtained from 4000–400 cm^−1^ with 64 scans. Origin software was employed for the process of peak splitting and the detailed analysis of the infrared spectrum.

## 5. Conclusions

To elucidate the phase transition point of E-Wc across varying volume ratios, a highly sensitive colorimetric assay based on starch–I_2_ crystallization was developed in this study. The results visually demonstrate two distinct transition points, occurring at volume ratios of 40–45 vol% and 75–77 vol% for E-Wc. The first transition is characterized by the disappearance of the characteristic blue coloration, while the second is marked by a significant alteration in the absorption intensity of the starch–I_2_ complex. Mechanistic investigations reveal that the supramolecular structure of E-Wc, rather than individual ethanol or water molecules within the binary mixture, governs the starch–I_2_ crystallization behavior. By employing medium-regulated starch–I_2_ crystallization as an analytical platform, we establish a direct correlation between the phase transition of E-Wc and the iodine chromogenic effect. This approach provides a novel and accessible optical probing strategy for investigating the transitions of colorless supramolecular assemblies. The unique value of the starch–iodine system as a cluster environment probe lies in its sensitivity to disturbances in the hydrogen bond network, which provides a new idea for the development of in situ detection techniques.

## Figures and Tables

**Table 1:** The ratios of absorbance between 1047, 1022, and 995 cm^−1^ after deconvolution of infrared spectra.

Name	ν_1_ (cm^−1^)	A_1_	ν_2_ (cm^−1^)	A_2_	ν_3_ (cm^−1^)	A_3_	A_1_/A_2_	A_2_/A_3_	R^2^
Native PS	1056	4.425	1021	9.018	980	9.989	0.4907	0.9028	0.9989
75%PS	1048	9.255	1015	7.034	980	11.98	1.316	0.5871	0.9988
77%PS	1054	7.378	1017	9.606	980	10.35	0.7681	0.9281	0.9994
Native PTS	1052	4.627	1024	6.274	989	11.85	0.7375	0.5295	0.9990
75%PTS	1051	8.327	1023	5.785	990	14.59	1.439	0.3965	0.9993
77%PTS	1053	6.927	1026	9.095	990	17.50	0.7616	0.5198	0.9990

## Data Availability

Data are contained within the article.

## Abbreviations

The following abbreviations are used in this manuscript:

E-Wc	Ethanol–water cluster(s)
E-Ws	Ethanol–water solution(s)
starch-I_2_	starch–iodine (system)
HB(s)	hydrogen bonding(s)
I_2_	iodine molecules
I_3_^−^	triiodide ions
PS	pea starch
PTS	potato starch
I_2_/I^−^	iodine/iodide solution
RT	room temperature
HPH	high-pressure homogenization
HK	honokiol
AC	arctin
XRD	X-ray diffraction
RC	relative crystallinity
SEM	scanning electron microscopy
FT-IR	Fourier transform infrared
